# Impact of depression and anxiety on health-related quality of life changes over time within individuals with rheumatoid arthritis or inflammatory bowel disease: A prospective Canadian cohort study

**DOI:** 10.1371/journal.pone.0349140

**Published:** 2026-05-28

**Authors:** Carol A. Hitchon, Charles N. Bernstein, James M. Bolton, Casandra Dolovich, Renee El-Gabalawy, Lesley A. Graff, Lisa M. Lix, James J. Marriott, John D. Fisk, RuthAnn Marrie

**Affiliations:** 1 Department of Internal Medicine, Max Rady College of Medicine, Rady Faculty of Health Sciences, University of Manitoba, Winnipeg, Manitoba, Canada; 2 Department of Psychiatry, Max Rady College of Medicine, Rady Faculty of Health Sciences, University of Manitoba, Winnipeg, Manitoba, Canada; 3 Department of Clinical Health Psychology, Max Rady College of Medicine, Rady Faculty of Health Sciences, University of Manitoba, Winnipeg, Manitoba, Canada; 4 Department of Community Health Sciences, Max Rady College of Medicine, Rady Faculty of Health Sciences, University of Manitoba, Winnipeg, Manitoba, Canada; 5 Department of Medicine, University of Toronto, St. Michael’s Hospital, Toronto, Ontario, Canada; 6 Nova Scotia Health and the Departments of Psychiatry, Psychology & Neuroscience, and Medicine, Dalhousie University, Halifax, Nova Scotia, Canada; Taipei Veterans General Hospital, TAIWAN

## Abstract

**Objectives:**

In individuals with rheumatoid arthritis (RA), inflammatory bowel disease (IBD), or primary depressive or anxiety disorders without RA or IBD (DEP/ANX) we aimed to evaluate between-person and within-person changes in physical and mental health-related quality of life (HRQoL) over time. We also aimed to compare the impacts of depression and anxiety symptoms on HRQoL, and to examine the roles of physical and cognitive functioning, fatigue, physical comorbidities, and disease activity, on HRQoL over time.

**Methods:**

As part of a single centre prospective cohort study, individuals with RA (n = 154), IBD (n = 247), or DEP/ANX (n = 306) recruited between November 2014 and July 2016 were seen annually for 3 years. Participants reported symptoms of depression and anxiety (Hospital Anxiety and Depression Scale), fatigue (Daily Fatigue Impact Scale), HRQoL (RAND-36), and completed functional assessments (physical: nine-hole peg test, timed 25-foot walk test; cognition: Symbol Digit Modalities Test). Generalized linear models with generalized estimating equations tested between-person and within-person associations of depression and anxiety with HRQoL in covariate-adjusted models that included socio-demographic characteristics, health status and medication use. Physical (PCS-36) and mental (MCS-36) HRQoL were assessed separately and comparatively for RA, IBD and DEP/ANX.

**Results:**

RA participants were older than IBD or DEP/ANX participants [mean age = 59.49(11.66), 47.45(14.80), 43.87(12.94)]. Most participants (>85%) reported meaningful changes in HRQoL. After adjustment, within-person increased depressive and anxiety symptoms were associated with reduced MCS-36 [depression: −7.91 (−9.45, −6.36), anxiety: −5.62 (−6.85, −4.39)]. Increased fatigue and worse cognition were associated with reduced PCS-36 [−0.33 (−0.38, −0.28); −0.31 (−0.64, 0.022)]. After adjustment, increased physical function, IBD or DEP/ANX diagnosis were associated with higher PCS-36 [1.47 (0.63, 2.30); 2.84 (1.60, 4.08); 5.23 (3.92, 6.55)].

**Conclusions:**

Variations in depression, anxiety, fatigue, cognition, and physical function are associated with HRQoL fluctuations in people with RA, IBD and DEP/ANX, highlighting the importance of addressing these issues while treating disease.

## Introduction

Rheumatoid Arthritis (RA) and Inflammatory Bowel Disease (IBD) are chronic immune-mediated inflammatory diseases (IMIDs) that predominantly affect the joints (RA) or the gastrointestinal tract (IBD), yet often have multisystem involvement [1]. Treatment often involves use of similar immune modulating agents. Despite advances in treatment approaches, people with these conditions experience chronic pain and disability that fluctuates in severity over time. Comorbid medical conditions are common in both RA and IBD patients. In addition, mental disorders such as depression and anxiety have higher prevalence rates in RA and IBD patients than those in the general population [2,3]. Both depression and anxiety can adversely impact disease course and the overall health related quality of life (HRQoL) of patients with these IMIDs [4–10].

Depressive and anxiety symptoms may have differing impact on physical and mental HRQoL, and these effects may vary across diseases. Additionally, variations in severity of mental health symptoms for an individual over time may also lead to variations in HRQoL over time. Information regarding this may help inform the design and timing of effective interventions to improve mental health in individuals with IMIDs. While prior reports have documented the association of comorbid depression and anxiety with HRQoL in people with RA or IBD, most studies have been cross-sectional, few have compared HRQoL across IMIDs, and the limited longitudinal studies mostly reported group averages [4,5,7,11–16]. Few studies have examined and compared within-person changes in mental health and HRQoL for patients with RA, IBD or primary depressive and anxiety disorders (DEP/ANX).

We evaluated changes in HRQoL and the impact of changes in depressive and anxiety symptoms on physical and mental HRQoL, at the individual level, in persons diagnosed with RA, IBD, or DEP/ANX who were followed annually over three years. The effects of fatigue [6,12], disease activity [15], physical and cognitive function and comorbidity burden [17] were also evaluated since these may fluctuate in severity and may contribute to changes in HRQoL in RA and IBD [9,18–21].

## Materials and methods

### Study population

Adults with physician-diagnosed RA, IBD or DEP/ANX were recruited between November 1 2014 and July 31 2016 as part of a prospective cohort study that examined psychiatric comorbidity in people with IMIDs residing in Manitoba, Canada [22]. Participants were recruited from outpatient gastroenterology, and rheumatology clinics and from research databases affiliated with these clinics, as well as from outpatient psychiatry clinics/groups. Some participants were recruited from primary care and posters in community settings. Participants completed questionnaires and underwent physical and cognitive assessments at baseline and annually for three years following enrollment (total of 4 visits). A total of 707 participants were followed. All participants provided written informed consent. The University of Manitoba Health Research Ethics Board approved the study (2014:201).

### Sample size

To test the baseline association between psychiatric comorbidity and HRQOL in each group, we assumed that at least 30% of the sample would experience any psychiatric comorbidity, confounders will explain 10% of the variation in the data, a 10% difference in HRQoL (MCS-36/PCS-36), a pooled standard deviation of 10, and alpha = 0.05. For longitudinal analyses, we assumed an annual attrition rate of 3%, an average annual rate of decline of 3% in HRQoL, a pooled between-group variance of 10, and a pooled within-group variance of 5. On the basis of these assumptions, each IMID group would need a minimum sample size of 150 [22].

### Health-related quality of life

We used the RAND-36 to evaluate physical and mental HRQoL. The RAND-36 has established population normative values and has been validated for use in studies of multiple chronic conditions thereby facilitating cross-disease comparisons [23]. The RAND-36 generates aggregate scores summarizing physical HRQoL (Physical Component Score, PCS-36) and mental HRQoL (Mental Component Score, MCS-36). Aggregate scores range from 0 to 100, with a mean of 50 and standard deviation of 10; higher scores represent better HRQoL.

### Symptoms and comorbidity

Symptoms of depression and anxiety were evaluated using the Hospital Anxiety and Depression Scale (HADS). The HADS includes 7 items for depression (HADS-D) and 7 items for anxiety (HADS-A) and has been validated in medical and psychiatric populations [24–26]. Total scores on each scale range from 0 to 21. Recommendations regarding the optimal cut-point for the HADS vary in IBD and RA. Thus, HADS scores were dichotomized using the more specific cut-point of ≥11 to indicate clinically meaningful elevations in symptoms of depression and anxiety; sensitivity analyses included these scores as continuous variables (1-point change). We evaluated fatigue using the eight-item Daily Fatigue Impact Scale (DFIS) [27], which has good psychometric properties in immune mediated diseases [28,29]. Scores range from 0 to 32; higher scores indicate more fatigue. Participants reported physician-diagnosed comorbidities using a validated questionnaire [30], which included diseases of the endocrine, cardiovascular, peripheral vascular, pulmonary, gastrointestinal, renal, and neurological systems, malignancy and other rheumatic conditions. Individuals with RA and IBD who have more comorbidities report lower HRQoL or worse outcomes; we categorized the number of comorbidities as 0, 1, 2 and ≥3 [17,31,32].

### Physical and cognitive function

Generic measures were used to assess physical function and cognition to allow disease comparisons. Lower limb function was assessed using the Timed 25-foot walk test, and upper limb function was assessed using the nine-hole peg test [33,34]. The Timed 25-foot walk test is a validated measure of functional capacity in older individuals and the nine-hole peg test is a validated measure in the Arthritis Hand Index [35–37]. We generated z-scores for lower and upper limb function using the baseline values of these measures for the cohort, then averaged them [38]. Cognitive function was assessed using the oral version of the Symbol Digit Modalities Test (SDMT) [39–41], a screening measure of processing speed; alternate SDMT forms were used at the second and fourth visits to limit practice effects. Lower scores indicate worse cognition. We used raw scores for the SDMT as we included age, gender, and education in our regression models, as described further below.

### RA and IBD disease activity

RA disease activity was recorded using the Clinical Disease Activity Index (CDAI) and active RA was defined as a CDAI score of >10 [42]. RA function was also assessed using the modified health assessment questionnaire (mHAQ) [43]. IBD activity was evaluated using the Harvey Bradshaw or Powel Tuck clinical indices (HB/PT) for Crohn’s disease and ulcerative colitis respectively; active symptomatic disease was defined as a HB/PT index of ≥5 [44].

### Covariates

Time-invariant covariates included self-reported gender, ethnicity, education, and age at symptom onset. As described elsewhere [22], participants reported ethnicity using the categories adopted by Statistics Canada in national surveys; responses were then categorized as White and Other as most self-identified as the former. Highest level of education attained was summarized as less than high school, high school, and some post-secondary education.

Time-varying covariates updated at each visit included age, annual household income, marital status, smoking status, body mass index (BMI), physical comorbidity count, physical function, cognitive function, disease activity (classified as active/not active), and use of disease-modifying therapy (DMT) (classified as yes/no). DMT included conventional and biologic immunomodulating drugs (Supplemental Table 1). Participants reported date of birth from which we derived age based on visit date. Consistent with our prior work, annual household income was categorized as <$50,000, ≥ $50,000, or ‘I do not wish to answer’. Marital status was classified as single (single/never married, divorced, widowed, separated) or partnered (married, common-law). We classified participants as current, past, and never smokers; smokers had smoked ≥100 cigarettes in their lifetime. We determined body mass index (BMI) (kg/m^2^) based on measured height and weight and categorized it as low or normal (< 25), overweight (≥25 to <30) or obese (≥30).

### Analysis

We included participants who had completed at least one visit; missing data were not imputed as only 5% were missing. We summarized participant characteristics using the mean, standard deviation (SD), median (interquartile range [IQR]), or frequency (percent) as appropriate. We summarized the PCS-36, MCS-36, HADS-D, HADS-A, fatigue, physical and cognitive function, and comorbidities at each visit. Since a 3-point change is considered clinically meaningful for the PCS-36 and MCS-36 [45–47], we also report the percentage of participants with ≥3-point increase or decrease in scores between visits. Generally, changes of 3–4 points represent small effects, > 4–10 points moderate effects, and >10 points large effects [48].

The two outcomes of interest were PCS-36 and MCS-36 (HRQoL). Each was evaluated separately, consistent with our prior work [38]. To determine the effect of the independent variables of interest on HRQoL we used generalized linear models with an identity link function and generalized estimating equations with an independent working correlation structure to fit sequential conditional mean models (SCMM) [49]. This approach allowed us to account for repeated measures and potential time-varying confounding. These generalized linear models produce outputs that are population averages of within-person and between-person effects. Since we were predominantly interested in within-person effects, we parameterized the model to disaggregate these effects by using person-specific mean centering to generate separate variables [50].

The primary independent variables of interest were the within-person variables for the HADS-D, and HADS-A. Between-person independent variables for the HADS-D and HADS-A are reported to enable comparisons with other published work. Secondary independent variables of interest were the within-person and between-person variables for physical function (z score, continuous), cognitive function, physical comorbidity count (none as reference) and disease activity (inactive as reference). As in our previous work we included fatigue (DFIS) as a predictor for the PCS-36 models but not the MCS-36 models because the MCS-36 captures fatigue/vitality whereas the PCS-36 does not [51]. We tested for two-way interactions between the within-person and between-person changes in depression and anxiety symptoms. To compare effects in RA, IBD, and DEP/ANX cohorts we included an independent variable for disease group (RA as reference). Unadjusted models included only the primary and secondary independent variables of interest. Adjusted models included the primary independent variables of interest (HADS-D, HADS-A) secondary independent variables of interest (physical function, cognitive function, fatigue, physical comorbidity count and disease activity) and covariates [age (continuous), gender (woman as reference), education (<high school as reference), income (<$50,000 as reference), race (White as reference), marital status (single as reference), BMI (normal as reference), smoking status (never as reference), age at RA or IBD onset (continuous), DMT use (none as reference)]. Separate disease specific models including the primary and secondary variables of interest, and covariates were used to test for interactions between within-person effects and between-person effects for primary variables (HADS-D, HADS-A) on PCS-36 and MCS-36.

Regression coefficients with 95% confidence intervals are reported for the association of independent variables with each HRQoL outcome. If the regression coefficient for a variable (e.g., HADS-D) is negative, then a 1 point increase in the variable (e.g., depression severity) is associated with a decrease in HRQoL. Based on the mean centering this also means that a 1 point decrease in the variable (e.g., depression severity) would be associated with an improvement in HRQoL.

Alpha values of < 0.05 were considered significant. Analyses were conducted using SAS V9.4 (SAS Institute Inc., Cary, NC).

### Patient and public involvement

A subgroup of participants provided perspectives on their educational needs relating to mental health and preferences for information dissemination through a web-based survey in which questions were partly informed by advisory groups consisting of patient organizations for RA, IBD or mental health disorders and patients with these conditions [22,52].

## Results

Table 1 shows the baseline demographics and clinical characteristics of participants [154 RA, 247 IBD (62% with Crohn’s disease), 306 DEP/ANX]. Most participants were women [521/707 (73.69%)]. RA participants were older than IBD and DEP/ANX participants [years of age at baseline mean (SD) RA 59.49(11.66), IBD 47.45(14.80), DEP/ANX 43.87 (12.94)). Most participants self-disclosed being of White ethnicity and over 65% had completed high school. Three-quarters of RA participants were on DMT, and at baseline, one-third had active RA [mean (SD) mHAQ = 4.20(4.08)]. Half of IBD participants were on DMT and 43% had active disease. Most DEP/ANX participants (n = 144, 93.51%) were receiving medication. Over one-third of RA and IBD participants had a life-time reported history of major depressive disorder (RA 38%, IBD 40%) and nearly one-third had a lifetime history of anxiety disorder (RA 31%, IBD 26%) (Supplemental Table 2).

**Table 1:** Participant characteristics.

Characteristic	RA	IBD	DEP/ANX	p-value^1^	p-value^2^	p-value^3^
N	154	247	306			
**Age**, yr mean (SD)	59.49 (11.66)	47.45 (14.80)	43.87 (12.94)	<.0001	<.0001	<.0001
**Gender**, Woman (%)	131 (85.1)	156 (63.2)	234 (76.5)	<.0001	<.0001	<.0001
**Ethnicity**, n (%)				0.4437	0.0117	0.4437
White	116 (75.3)	210 (85.4)	245 (80.1)			
Aboriginal	11 (7.1)	13 (5.3)	22 (7.2)			
Other	27 (17.5)	23 (9.4)	39 (12.8)			
**Education**, n (%)				0.0695	<.0001	0.0097
High school or less	50 (33.6)	76 (30.9)	101 (33.7)			
College/Technical/Trade	61 (40.9)	81 (32.9)	105 (35.0)			
University bachelors or higher	38 (25.5)	89 (36.2)	94 (31.3)			
**Annual income**, n (%)				0.70	<.0001	<.0001
<$50,000	70 (45.5)	58 (23.5)	142 (46.4)			
>=$50,000	73 (47.4)	171 (69.2)	139 (45.4)			
I do not wish to answer	11 (7.1)	18 (7.3)	25 (8.2)			
**Marital Status**, n (%)				<.0001	<.0001	<.0001
Single/never married	19 (12.3)	56 (22.7)	110 (36.0)			
Married/common law	93 (60.4)	160 (64.8)	133 (43.5)			
Divorced/separated/widowed	42 (27.3)	31 (12.6)	63 (20.6)			
**Smoking status**, n (%)				<.0001	0.4769	<.0001
Never smoker	59 (38.3)	108 (43.7)	151 (49.4)			
Past smoker	73 (47.4)	97 (39.3)	91 (29.7)			
Current smoker	22 (14.3)	42 (1.0)	64 (20.9)			
**Weight (kg), n (%)**				0.0006	0.0030	<.0001
BMI < 25	53 (34.42)	96 (38.87)	95 (31.05)			
BMI 25 to <30	47 (30.52)	87 (35.22)	82 (26.80)			
BMI ≥ 30	54 (35.06)	64 (25.91)	129 (42.16)			
**Current use of any DMT n (%)**	144 (93.51)	185 (74.90)	–	–	<.0001	–

RA = rheumatoid arthritis, IBD = inflammatory bowel disease, DEP/ANX = primary depression or anxiety SD = standard deviation, BMI = body mass index, HADS = Hospital Anxiety and Depression Scale, MDD = major depressive disorder, SDMT = Symbol Digit Modalities Test, 9HPT = nine hole peg test Physical functioning z-score which is an average of the z-score for the timed 25-foot walk and nine-hole peg test, BMI = body mass index, DMT = disease modifying therapy.

^1^RA versus DEP/ANX; ^2^RA vs IBD; ^3^IBD versus DEP/ANX.

Most participants completed all four visits [RA n = 129 (83.8%), IBD n = 214 (86.6%), DEP/ANX n = 246 (80.4%)], however 5 (3.3%) RA, 7 (2.8%) IBD, and 20 (6.5%) DEP/ANX participants completed only a single visit. Over the study, 3 RA, 7 IBD and 3 DEP/ANX patients died; the remaining non-completers were lost to follow-up. At baseline, RA non-completers were more likely to have active RA, IBD non-completers were more likely to report anxiety and fatigue, and DEP/ANX non-completers were more likely to be young and report fatigue (Supplemental Table 3).

Mean (SD) PSC-36 and MCS-36 scores remained lower for RA, IBD and DEP/ANX than population normative values of 50 over the course of the study. For RA and IBD, on average, HRQoL scores, mean (SD) HADS- scores for depression and anxiety, fatigue, cognition, and physical function did not change significantly over the course of the study. DEP/ANX participants had more variable MCS-36, HADS-D and HADS-A, DFIS, SDMT and physical function scores although their PCS-36 scores did not change significantly (Supplemental Table 4).

**Fig 1:**
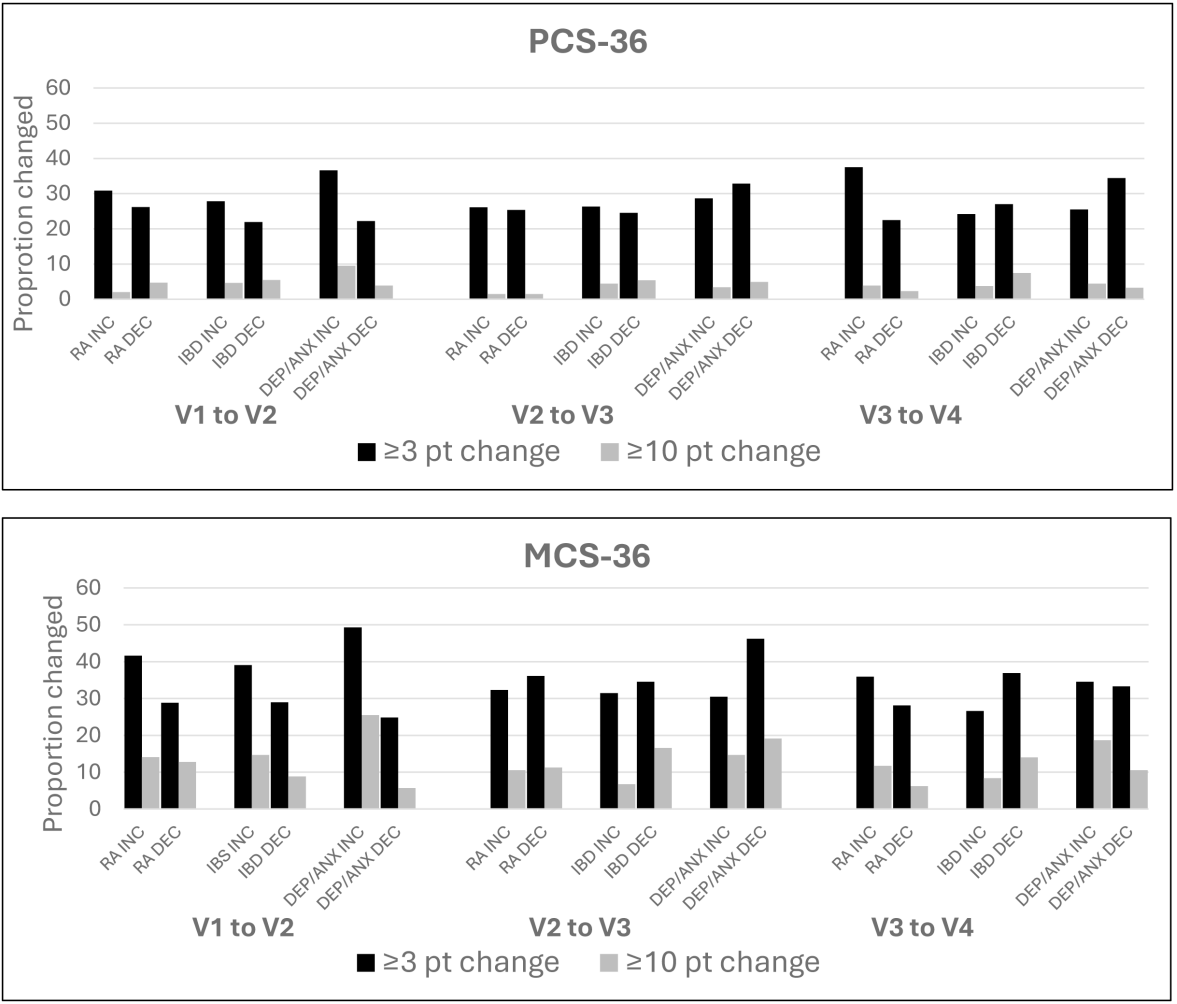
Proportion of participants reporting a change in health-related quality of life between visits. PCS = physical composite score, MCS = mental composite score, RA = rheumatoid arthritis, IBD = inflammatory bowel disease, DEP/ANX = primary depression or anxiety. Any change considered a ≥ 3-point difference on PCS-36 or MCS-36. Large change considered a ≥ 10-point difference on PCS-36 or MCS-36.

We examined the proportion of participants with at least a 3-point change in PCS-36 or MCS-36 scores between visits regardless of direction. Most participants reported meaningful changes in HRQoL at some point in the study period [RA PCS-36 N = 135 (87.66%), MCS-36 N = 143 (92.86%); IBD PCS-36 N = 209 (84.62%), MCS-36 N = 223 (90.28%); DEP/ANX PCS-36 N = 260(84.97%), MCS-36 N = 278 (90.85%)]. For RA participants, between any time points, PCS-36 improved for 104 (67.53%) and declined for 88 (57.14%); MCS-36 improved for 118 (76.62%) and declined for 110 (71.43%). For IBD participants, between any time points, PCS-36 improved for 162 (65.59%) and declined for 152 (61.54%); MCS-36 improved for 180 (72.87%) and declined for 179 (72.47%). For DEP/ANX participants between any time points PCS-36 improved for 216 (70.59%) and declined for 195 (63.73%); MCS-36 improved for 234 (76.47%) and declined for 214 (69.93%) (Fig 1).

Over the study nearly one-third of participants reported a large change (± > 10 points) in PCS-36 (RA 24.03%, IBD 31.17%, DEP/ANX 29.08%) while nearly half reported a large change in MCS-36 (RA 48.05%, IBD 52.63%, DEP/ANX 65.03%). A large increase in PCS-36 was reported by 12.99% RA, 15.76% IBD, and 18.63% DEP/ANX participants whereas a large decrease in PCS-36 was reported by 12.34% RA, 21.05% IBD, and 16.99% DEP/ANX participants. A large increase in MCS-36 was reported by 35.06% RA, 29.96% IBD and 50.33% DEP/ANX participants, while a large decrease in MCS-36 was reported by 27.27% RA, 38.87% IBD and 35.95% DEP/ANX participants (Fig 1).

In regression models adjusted for covariates, over the three years, RA participants with elevated symptoms of anxiety, fatigue, worse physical function (z arm/leg scores), worse cognitive function, active disease, and more comorbidities, had on average, lower PCS-36 scores. RA participants with elevated symptoms of depression and anxiety and active disease had, on average, lower MCS-36 scores (Table 2). After adjusting for covariates, IBD participants had, on average, lower PCS-36 scores in the presence of higher fatigue, active disease, and more comorbidities. IBD participants had, on average, lower MCS-36 scores with elevated symptoms of depression, anxiety, and active disease. HRQoL scores were not affected by IBD subtype (Table 3).

**Table 2:** Rheumatoid Arthritis Regression models for contributors to health-related quality of life.

Outcome	HRQOL -RA unadjusted models		HRQoL -RA adjusted models	
	PCS-36	MCS-36	PCS-36	MCS-36
	(95% CI)	(95% CI)	(95% CI)	(95% CI)
N obs. in model	569	568	518	517
BPC HADS-D ≥ 11	**−13.29 (−17.31, −9.26)**	**−27.73 (−32.86, −22.60)**	2.77 (−2.06, 7.62)	**−15.08 (−20.59, −9.57)**
WPC HADS-D ≥ 11	**−0.88 (−2.93, 1.16)**	**−7.96 (−11.40, −4.53)**	0.58 (−1.03, 2.20)	**−6.30 (−9.81, −2.79)**
BPC HADS-A ≥ 11	**−12.46 (−16.02, −8.89)**	**−24.78 (−29.58, −19.98)**	**3.90 (−0.31, 8.11)**	**−14.21 (−19.08, −9.34)**
WPC HADS-A ≥ 11	**−0.89 (−2.45, 0.68)**	**−4.64 (−7.21, −2.07)**	0.045 (−1.51, 1.60)	**−3.11 (−5.79, −0.43)**
BPC in DFIS	**−0.88 (−1.01, −0.75)**	**−1.31 (−1.44, −1.18)**	**−0.71 (−0.91, −0.50)**	
WPC DFIS	**−0.32 (−0.43, −0.22)**	**−0.66 (−0.83, −0.48)**	**−0.29 (−0.40, −0.18)**	
BPC zarm_leg	**5.17 (3.15, 7.18)**	**4.44 (2.59, 6.30)**	**2.39 (0.60, 4.18)**	1.37 (−0.63, 3.36)
WPC zarm_leg	**2.72 (1.73, 3.72)**	**3.11 (0.74, 5.48)**	**2.37 (1.19, 3.56)**	**2.76 (0.84, 4.68)**
BPC in SDMT	**1.73 (0.30, 3.15)**	**2.26 (0.77, 3.77)**	**−1.10 (−2.00, −0.189)**	−0.42 (−1.70, 0.86)
WPC SDMT	−0.33 (−1.13, 0.47)	1.06 (−0.31, 2.43)	−0.47 (−1.28, 0.34)	0.52 (−0.82, 1.87)
BPC CDAI >10	**−13.86 (−17.01, −10.71)**	**−14.36 (−19.02, −9.71)**	**−4.23 (−6.64, −1.82)**	**−5.02 (−8.58, −1.46)**
WPC CDAI >10	**−2.23 (−3.39, −1.07)**	**−1.89 (−3.55, −0.23)**	**−1.70 (−2.88, −0.52)**	**−1.53 (−3.16, 0.11)**
BPC comorbidities	**−1.33 (−1.96, −0.71)**	−0.61 (−1.42, 0.20)	**−0.77 (−1.22, −0.31)**	−0.11 (−0.71, 0.49)
WPC comorbidities	−0.30 (−1.17, 0.56)	0.87 (−0.55, 2.29)	−0.19 (−1.00, 0.62)	0.90 (−0.46, 2.26)

RA = rheumatoid arthritis, PCS = physical composite score; MCS = mental composite score, N obs. = number of observations used in model; BPC = between person change in variable; WPC = within person change in variable; HADS = Hospital Anxiety and Depression Scale D = Depression, A = Anxiety, DFIS = daily fatigue impact scale, Zarm_leg = Physical functioning z-score which is an average for the z-score for the timed 25-foot walk and nine-hole peg test, SDMT = Symbol Digit Modalities Test, CDAI = Clinical Disease Activity Index >10 for disease activity; comorbidities = number of comorbidities (0, 1, 2, ≥ 3).

Unadjusted model: Independent variables, no covariates; Adjusted models: Independent variables + covariates; adjusted for age at RA or IBD onset (continuous), gender (woman as reference), education, high school as reference), income 9,$50,000 as reference), race (White as reference), smoking status (never as reference), marital status (single as reference), body mass index (normal as reference), disease modifying therapy (none as reference).

No significant between-person and within person HADSA or HADSD interaction for PCS-36 or MCS-36.

Values in bold considered significant.

**Table 3:** Inflammatory Bowel Disease regression models for contributors to health-related quality of life.

	HRQOL -IBD unadjusted models		HRQOL -IBD adjusted models	
Outcome	PCS-36	MCS-36	PCS-36	MCS-36
	(95% CI)	(95% CI)	(95% CI)	(95% CI)
N obs. in model	924	924	844	844
BPC HADS-D ≥ 11	**−19.36 (−23.59, −15.13)**	**−34.97 (−39.85, −30.09)**	−1.58 (−5.24, 2.07)	**−13.99 (−19.60, −8.38)**
WPC HADS-D ≥ 11	**−3.66 (−6.26, −1.06)**	**−8.96 (−12.71, −5.21)**	−0.74 (−3.29, 1.81)	**−8.66 (−12.05, −5.28)**
BPC HADS-A ≥ 11	**−11.73 (−15.02, −8.44)**	**−22.22 (−25.83, −18.60)**	−0.49 (−3.23, 2.25)	**−15.33 (−18.88, −11.78)**
WPC HADS-A ≥ 11	**−1.97 (−3.26, −0.68)**	**−7.16 (−9.53, −4.79)**	−0.70 (−1.80, 0.40)	**−5.76 (−8.05, −3.47)**
BPC DFIS	**−0.97 (−1.10, −0.85)**	**−1.27 (−1.41, −1.14)**	**−0.65 (−0.78, −0.52)**	
WPC DFIS	**−0.42 (−0.49, −0.34)**	**−0.78 (−0.91, −0.64)**	**−0.34 (−0.42, −0.27)**	
BPC zarm_leg	**6.26 (4.29, 8.24)**	4.52 (2.09, 6.94)	1.23 (−0.63, 3.09)	2.00 (−0.84, 4.84)
WPC zarm_leg	3.60 (1.88, 5.31)	2.14 (−0.37, 4.65)	**2.74 (0.89, 4.58)**	0.95 (−1.52, 3.43)
BPC SDMT	**1.97 (1.04, 2.91)**	1.34 (0.12, 2.56)	0.08 (−0.51, 0.68)	0.39 (−0.66, 1.45)
WPC SDMT	0.36 (−0.13, 0.86)	0.07 (−0.71, 0.85)	0.095 (−0.36, 0.55)	0.28 (−0.83, 1.38)
BPC HB-PT ≥ 5	**−13.18 (−15.41, −10.96)**	**−11.81 (−15.25, −8.38)**	**−6.08 (−7.71, −4.45)**	**−4.99 (−7.63, −2.34)**
WPC HB-PT ≥ 5	**−3.47 (−4.53, −2.40)**	−3.38 (−5.15, −1.61)	**−2.93 (−3.85, −2.01)**	**−2.85 (−4.63, −1.07)**
BPC comorbidities	**−1.76 (−2.26, −1.26)**	**−0.79 (−1.44, −0.12)**	**−0.99 (−1.40, −0.59)**	−0.53 (−1.18, 0.11)
WPC comorbidities	0.21 (−0.70, 1.12)	0.23 (−0.95, 1.42)	0.17 (−0.50, 0.84)	0.36 (−0.81, 1.53)
CD vs UC	**–**	**–**	−0.70 (−1.78, 0.37)	−0.52 (−2.39, 1.35)

IBD = inflammatory bowel disease, CD = Crohn’s disease, UC = ulcerative colitis, N obs. = number of observations used in model; PCS = physical composite score; MCS = mental composite score, BPC = between person change in variable; WPC = within person change in variable; HADS = Hospital Anxiety and Depression Scale D = Depression, A = Anxiety, DFIS = daily fatigue impact scale, Zarm_leg = Physical functioning z-score which is an average for the z-score for the timed 25-foot walk and nine-hole peg test, SDMT = Symbol Digit Modalities Test; HB-PT = Harvey Bradshaw Patient Index ≥5 for symptomatic disease activity; comorbidities = number of comorbidities (0, 1, 2, ≥ 3).

Unadjusted models include independent variables, no covariates; Adjusted models include: Primary (HADS-D, HADS-A) and secondary (physical function, cognitive function, fatigue, physical comorbidity count and disease activity) independent variables + covariates; adjusted for (continuous), age at RA or IBD onset (continuous), gender (woman as reference), education,high school as reference), income 9,$50,000 as reference), race (White as reference), smoking status (never as reference), marital status (single as reference), body mass index (normal as reference), disease modifying therapy (none as reference). No significant between-person and within-person interactions for HADS for PCS-36 or MCS-36. Values in bold considered significant.

We were particularly interested in factors affecting change in HRQoL within individuals over time. Within individuals with RA, PCS-36 scores were lower (worsened) with increased fatigue, poorer physical function, and active disease. In contrast, MCS-36 scores were lower (worsened) with clinically meaningfully elevated symptoms of depression, anxiety, poorer physical function, and active disease (Table 2). Sensitivity analysis in which HADS was a continuous variable in the RA models showed that a one-point increase in HADS-D scores was associated with lower PCS-36 (by 0.45) and MCS-36 (by 0.78) scores and one point increase in HADS-A was associated with lower MCS-36 (by 0.75) scores (Supplemental Table 5). Within individuals with IBD, PCS-36 scores were lower (worsened) with increases in fatigue, poorer physical function, and active disease whereas MCS-36 scores were lower (worsened) with increased symptoms of depression and anxiety, and active disease (Table 3). Sensitivity analysis in which HADS was a continuous variable in the IBD models showed that one-point increases in HADS scores were associated with lower PCS-36 (by 0.28) and MCS-36 (by 1.45) scores, and one-point increases in HADS-A were associated with lower MCS-36 (by 0.75) scores (Supplemental Table 6). One point changes in scores for fatigue, physical and cognitive function, and having active disease also impacted PCS-36 and MCS-36 scores for individuals with RA or IBD. Similar findings were seen for individuals with DEP/ANX (with HADS scores ≥11) (Supplemental Table 7). In disease-specific regression models, we observed a less than additive effect when we tested for interactions of within-person effects and between-person effects for HADS-D and PCS-36 in RA (0.08 (0.01, 0.15) but not for HADS-A and not in IBD (Supplemental Table 8).

Regression models, adjusted for covariates and testing for cross-disease comparisons showed that on average, RA participants had poorer PCS-36 scores than IBD or DEP/ANX participants, whereas MCS-36 scores were better among RA participants than among DEP/ANX participants. IBD participants also had worse PSC-36 scores but better MSC-36 scores than DEP/ANX participants and their MCS-36 scores were similar to RA participants (Table 4). Regression models including all three conditions found less than additive effects when testing for interactions for HADS-D (as a continuous variable) for both PCS-36 and MCS-36 scores [PCS-36 0.05(0.02, 0.08) and MCS-36 0.10(0.05,0.14)], but not for HADS-A (Supplemental Table 8).

**Table 4:** Regression models comparing disease groups.

Outcome	HRQOL-unadjusted model		HRQOL-adjusted model	
**PCS-36**	**MCS-36**	**PCS-36**	**MCS-36**
**(95% CI)**	**(95% CI)**	**(95% CI)**	**(95% CI)**
N obs. in model	2464	2462	2464	2462
BPC HADS-D ≥ 11	**−12.50 (−14.63, −10.38)**	**−28.58 (−30.51, −26.65)**	−1.13 (−3.27, 1.01)	**−16.95 (−19.00, −14.90)**
WPC HADS-D ≥ 11	**−2.24 (−3.26, −1.22)**	**−9.24 (−10.89, −7.58)**	−0.79 (−1.71, 0.13)	**−7.91 (−9.45, −6.36)**
BPC in HADS-A ≥ 11	**−6.55 (−8.25, −4.85)**	**−21.67 (−23.35, −19.98)**	0.66 (−0.94, 2.27)	**−12.31 (−14.13, −10.49)**
WPC HADS-A ≥ 11	**−1.46 (−2.14, −0.80)**	**−6.23 (−7.54, −4.92)**	−0.49 (−1.10, 0.12)	**−5.62 (−6.85, −4.39)**
BPC in DFIS	**−0.80 (−0.87, −0.73)**		**−0.71 (−0.80, −0.63)**	
WPC DFIS	**−0.35 (−0.40, −0.31)**		**−0.33 (−0.38, −0.28)**	
BPC in zarm_leg	**6.98 (5.75, 8.20)**	**2.89 (1.50, 4.28)**	**2.60 (1.42, 3.78)**	**1.81 (0.34, 3.37)**
WPC zarm_leg	**1.86 (1.02, 2.69)**	2.08 (0.72, 3.44)	**1.47 (0.63, 2.30)**	1.43 (0.29, 2.57)
BPC SDMT	2.29 (1.75, 2.83)	**1.28 (0.52, 2.03)**	−0.24 (−0.71, 0.24)	**0.04 (−0.52, 0.60)**
WPC SDMT	−0.047 (−0.41, 0.32)	0.76 (0.11, 1.41)	**−0.31 (−0.64, 0.022)**	0.25 (−0.34, 0.84)
BPC comorbidities	**−1.86 (−2.14, −1.59)**	−0.50 (−0.92, −0.09)	**−1.05 (−1.30, −0.79)**	−0.35 (−0.69, −0.03)
WPC comorbidities	−0.33 (−0.80, 0.13)	0.41 (−0.30, 1.12)	−0.26 (−0.68, 0.15)	0.27 (−0.41, 0.95)
**Disease Cohort**				
RA	ref	ref	ref	ref
DEP/ANX	**5.02 (3.28, 6.76)**	−8.95 (−11.09, −6.81)	**5.23 (3.92, 6.55)**	**−2.64 (−4.46, −0.83)**
IBD	**7.01 (5.24, 8.78)**	1.42 (−0.78, 3.63)	**2.84 (1.60, 4.08)**	−0.20 (−1.95, 1.54)

RA = rheumatoid arthritis; IBD = inflammatory bowel disease; DEP/ANX = primary depression or anxiety; PCS = physical composite score; MCS = mental composite score, HADS = Hospital Anxiety and Depression Scale D = Depression, A = Anxiety, DFIS = daily fatigue impact scale, SDMT = Symbol Digit Modalities Test.

Unadjusted models include independent variables, no covariates; Adjusted models include: Primary (HADS-D, HADS-A) and secondary (physical function, cognitive function, fatigue, physical comorbidity count and disease activity) independent variables + covariates; adjusted for (continuous), age at RA or IBD onset (continuous), gender (woman as reference), education,high school as reference), income 9,$50,000 as reference), race (White as reference), smoking status (never as reference), marital status (single as reference), body mass index (normal as reference), disease modifying therapy (none as reference). No significant between-person and within-person interactions for HADS for PCS-36 or MCS-36. Values in bold considered significant.

## Discussion

In this longitudinal study, we demonstrated reduced physical and mental HRQoL in people with diagnosed RA or IBD and identified factors that associate with HRQoL over time in individuals with these IMIDs as well as in individuals with DEP/ANX. Mental HRQoL reported by participants with RA was similar to that of participants with IBD, but better than that of participants with DEP/ANX after accounting for severity of symptoms of depression and anxiety. However, RA participants reported worse physical HRQoL than participants with either IBD or DEP/ANX. Within individuals, HRQoL fluctuated over time and was influenced by variations in symptoms of depression and anxiety as measured by the HADS. Among individuals with RA or IBD, increased clinically meaningful symptoms of depression and anxiety over time were associated with lower mental HRQoL, whereas even small increases in symptoms of depression were associated with lower physical HRQoL. Similarly, within individuals with RA, IBD or DEP/ANX, increased fatigue, poorer physical and cognitive function, and active disease (for RA and IBD) were associated with reduced physical HRQoL. These data support prior reports documenting, on average, people with RA and IBD have reduced HRQoL compared to population norms and that in cohorts of people with RA or IBD, HRQoL is impacted by both depression and anxiety [4–9] We provide new data on the magnitude of these effects for individuals over time.

On a group level, HRQoL measures were stable over time, yet examining individual trajectories, there was considerable variability across time in both physical and mental HRQoL driven not only by disease activity, but also by changes in mental health symptoms, fatigue, cognition, and physical function. These within-individual HRQoL changes were clinically meaningful based on estimates in RA patients [53]. The observed discrepancy in significant associations found for group-based (between-person) versus individual-based (within-person) analyses highlights the importance of some of these factors to an individual and informs the magnitude of potential benefit of effective interventions to an individual. Thus, within-person effects are most relevant for clinical practice. Moreover, between-person effects may not apply at the individual level. As an example, we found comorbidity burden (RA and IBD) and cognition (RA) were associated with HRQoL when analysed on a group level but not when analyzed at the level of the individual. This may reflect relative stability of these variables over the limited timeframe of this study and points to the need for even longer duration studies with larger sample sizes.

Our study design enabled cross disease comparisons for RA and IBD, IMIDs with different inflammatory phenotypes but similar treatment in term of therapies targeting inflammation [1]. The effect of changes in symptoms of depression, anxiety, fatigue, and disease activity on HRQoL were similar for RA and IBD individuals, yet changes in physical function affected both physical and mental HRQoL for individuals with RA, but only physical HRQoL for individuals with IBD. This may be due to response shift that occurs when an individual reconceptualizes their HRQoL over time [54], may reflect differences in the sensitivity to change of the measures used to assess disease activity in these conditions, or may reflect differences in disease and demographic characteristics. RA articular flares can profoundly impact function which may lead to greater impact on both physical and mental HRQoL. This, or increased disease severity, may also explain why the RA participants had the poorest physical HRQoL across the conditions studied. Importantly, the main effects of variables on HRQoL observed when RA and IBD were analyzed separately were also seen in models comparing RA, IBD and DEP/ANX cohorts. Mental HRQoL was similar across RA, IBD and DEP/ANX further highlighting the cross-disease relevance of mental health. With regards to fatigue, RA and IBD had similar scores over time whereas persons with DEP/ANX had significantly higher levels of fatigue over time. This suggests that mental illness may contribute to greater fatigue impact on functioning than physical inflammatory diseases.

We evaluated the effects of depression and anxiety symptoms separately and assessed the effect of cognition on HRQoL. Targeted interventions for anxiety and depression symptoms can be quite different and it is important to distinguish these to guide management [55,56]. Cognition has not been commonly included in prior studies of HRQoL in RA and IBD. While there is growing interest in the presence of cognitive impairment in these conditions [57–59], and in the association of depressive and anxiety disorders with cognitive impairment in these disorders [60], the impact of cognitive impairment on HRQoL in these conditions has received little attention. The SDMT measures information processing speed including elements of visual scanning, attention and working memory which is known to affect instrumental activities of daily living such as medication management and cooking as well as driving and employment [40,61–63]. Here we found worse cognition as measured by the SDMT, was associated with reduced HRQoL in people with RA, IBD and ANX/DEP, illustrating the potential impacts of cognitive changes in these populations. Further study incorporating a more comprehensive assessment of cognition would clarify the role of changes in cognitive function and HRQoL.

Our study has several strengths including assessing individuals over time, separate analysis of physical and mental HRQoL and of anxiety and depression, analysis of cognition and fatigue symptoms which can overlap with both IMID disease and DEP/ANX, and cross-disease comparisons. We also acknowledge the study limitations. Despite employing multiple approaches to enable study participation by individuals from groups often underrepresented in studies, including broad recruitment strategies, flexible procedures for data collection and compensation for out-of-pocket expenses related to study participation, our population, while representative of many cohort studies of these IMIDs, was predominantly white, well-educated women. Thus, our findings may not be generalizable more broadly to individuals with RA, IBD and DEP/ANX. While we had very high rates of study completion and variable disease activity in participants, there were differences in baseline clinical activity between study completers and the few non-completers, potentially introducing selection bias. As expected for these conditions, age differed across cohorts and may have influenced age-related comorbidity, frailty, and related impacts on physical function. While age and comorbidity were controlled for in the analysis, and physical function specifically assessed, residual confounding could persist and we did not test for interactions between age and other variables. We were unable to specifically examine the impact of active extra-articular disease in RA participants or extra-intestinal disease in IBD participants on HRQoL outcomes as we lacked detailed phenotypic data. However, the composite RA CDAI activity index includes a patient reported global assessment of disease activity [42] and the composite IBD HB/PT activity indices capture active extra-intestinal features [44], including articular disease which has been shown to impact HRQoL in other studies [64]. We did not examine the role of corticosteroid treatment in fluctuations of HRQoL. Although our findings suggest that improved management of mental health symptoms can improve HRQoL over time, an outcome prioritized by patients, we lacked information about the treatments for mental health symptoms that participants were receiving, which could have allowed us to examine this issue. Similarly, while depression is associated with active IMID disease activity over time, [65] in this analysis we were unable to determine if improved treatment of disease activity with DMT impacts symptoms of depression or anxiety. We used a generic measure of HRQoL, rather than disease-specific measures to allow for comparisons across diseases while limiting participant response burden. However, disease-specific measures may have provided greater sensitivity to change and may have performed better than the RAND-36 in capturing elements important to HRQoL within the individual IMID cohorts. A similar measure, the Short Form-36 (SF-36), has been evaluated in recent systematic reviews of HRQoL in RA [4] and IBD [7,21]. The SF-36 and RAND-36 generally lead to similar scores across chronic medical conditions; however, the RAND-36 assesses mental health better than the SF-36 and is freely available [66,67]. Although within-individual minimally important clinical differences in SF-36 composite scores have been reported for RA [53], these data are not available for the RAND-36 nor for IBD. Similarly, we used general measures of physical function to assess upper and lower extremity function across diseases which are valid in other IMIDs with physical limitations and the general population [33–37]. HRQoL represents a broad construct and other variables with likely impacts such as resilience and self-efficacy were not assessed to maintain study feasibility. However, the within-person analysis controlled for these and other time-invariant factors.

For the clinician, the findings emphasize the importance of detection and management of symptoms of depression and anxiety. Interventions to improve mental health must be shown to be effective, sustainable, and cost-efficient before implementation. The observed fluctuations in the severity of depression and anxiety symptoms over time highlight the need for ongoing adaptable strategies to manage mental health and demonstrate the impact of even modest changes in symptom severity to HRQoL. Cost-effectiveness includes assessment of indirect costs such as HRQoL associated with an intervention. The results of this study can be used to estimate impact on HRQoL, and related indirect costs, that will occur based on the observed improvement in depression and anxiety symptoms achieved by an intervention. These data are needed before adoption and implementation of interventions, particularly in health systems with a public payer.

## Conclusions

We found that fluctuations in symptoms of depression and anxiety drive clinically significant variability in HRQoL for individuals with RA, IBD and DEP/ANX. Our findings support the relevance of screening for symptoms of depression and anxiety as part of the routine clinical evaluation of patients with these IMIDs. This can be achieved using tools that are simple and feasible for busy medical practices [25,26]. Further study is warranted to determine if interventions that target symptoms of depression and anxiety can improve HRQoL for individuals with RA or IBD.

### Strengths and limitations

Strengths:

- Assessed the impact of depression and anxiety on physical and mental Health-Related Quality of Life, cognition physical function and fatigue within individuals with immune mediated inflammatory diseases over a three-year period using validated self report measures.
- Included participants with Rheumatoid Arthritis, Inflammatory Bowel Disease, or primary depressive or anxiety disorders to enable cross-disease comparisons.
- Used analytical methods to enable evaluation of change over time in physical and mental Health-Related Quality of Life.

Limitations:

- Used general measures of Health-Related Quality of Life and physical function rather than disease-specific measures that may better capture disease-specific features.
- Limited participation of groups often underrepresented in research studies despite efforts to facilitate their study participation.

## Supporting information

Supplemental Table 1 Disease modifying treatment used in rheumatoid arthritis and inflammatory bowel disease participants. (DOCX)

Supplemental Table 2 Patient comorbidity, symptoms, and function at baseline stratified by disease group. RA = rheumatoid arthritis, IBD = inflammatory bowel disease, DEP/ANX = primary depression or anxiety, SD = standard deviation, BMI = body mass index, MDD = major depressive disorder, HADS = Hospital Anxiety and Depression Scale D = depression A = anxiety, SDMT = Symbol Digit Modalities Test, 9HPT = nine hole peg test Physical functioning z-score which is an average of the z-score for the timed 25-foot walk and nine-hole peg test. ^1^p-value for comparison of RA versus DEP/ANX; ^2^ p-value for comparison of RA versus IBD; ^3^ p = value for comparison of IBD versus DEP/ANX; p value < 0.05 in bold considered significant.
(DOCX)

Supplemental Table 3 Characteristics of study completers vs non-completers of all 4 visits stratified by disease group. RA = rheumatoid arthritis, IBD = inflammatory bowel disease, DEP/ANX = primary depression or anxiety, SD = standard deviation, BMI = body mass index, HADS = Hospital Anxiety and Depression Scale D = depression A = anxiety, MDD = major depressive disorder, SDMT = Symbol Digit Modalities Test, 9HPT = nine hole peg test Physical functioning z-score which is an average of the z-score for the timed 25-foot walk and nine-hole peg test, DMT = disease modifying treatment. ^1^IBD completers vs non-completers, ^2^RA completers vs non-completers, ^3^DEP/ANX completers vs non-completers, p value < 0.05 in bold considered significant.
(DOCX)

Supplemental Table 4 Health- related quality of life, symptoms and function across study visits. RA = rheumatoid arthritis; IBD = inflammatory bowel disease, DEP/ANX = depression and anxiety, PCS = physical composite score; MCS = mental composite score, HADS = Hospital Anxiety and Depression Scale D = Depression, A = Anxiety, DFIS = daily fatigue impact scale, SDMT = Symbol Digit Modalities Test, NHP = nine hole peg test, SD = standard deviation; V1 = first (baseline) visit, V2 = second visit (first annual follow-up), V3 = third visit (second annual follow-up), V4 = forth annual follow-up) visit.
(DOCX)

Supplemental Table 5 Rheumatoid Arthritis Regression models with continuous Hospital Anxiety and Depression Scale scores. RA = rheumatoid arthritis, PCS = physical composite score; MCS = mental composite score, HADS = Hospital Anxiety and Depression Scale D = Depression, A = Anxiety, DFIS = daily fatigue impact scale, SDMT = Symbol Digit Modalities Test, DMT = disease modifying therapy, Zarm_leg = Z-score for physical function. Unadjusted models: Independent variables, no covariates; Adjusted models: Independent variables + covariates (age (continuous), age at symptom onset (continuous), gender (woman as reference), education (<high school as reference), income (<$50,000 as reference), race (White as reference), smoking status (never as reference), marital status (single as reference), Body mass index (normal as reference), disease modifying therapy). Values in bold considered significant.
(DOCX)

Supplemental Table 6 Inflammatory Bowel Disease regression models with continuous Hospital Anxiety and Depression Scale scores. IBD = inflammatory bowel disease, CD = Crohn’s disease, UC = ulcerative colitis, PCS = physical composite score; MCS = mental composite score, HADS = Hospital Anxiety and Depression Scale D = Depression, A = Anxiety, DFIS = daily fatigue impact scale, SDMT = Symbol Digit Modalities Test. Unadjusted models: Independent variables, no covariates; Adjusted models include independent variables + covariates [age (continuous), age at symptom onset (continuous), gender(woman as reference), education (<high school as reference), income (<$50,000 as reference), race (White as reference), smoking status (never as reference), marital status (single as reference), body mass index (normal as reference), disease modifying therapy (none as reference)]. Values in bold considered significant.
(DOCX)

Supplemental Table 7 Depression and Anxiety Regression models for contributors to health-related quality of life. PCS = physical composite score; MCS = mental composite score, HADS = Hospital Anxiety and Depression Scale D = Depression, A = Anxiety, DFIS = daily fatigue impact scale, Zarm_leg = Physical functioning z-score which is an average for the z-score for the timed 25-foot walk and nine-hole peg test, SDMT = Symbol Digit Modalities Test Unadjusted models include independent variables, no covariates. Adjusted models include independent variables + covariates [age (continuous), age at symptom onset (continuous), gender (woman as reference), education (<high school as reference), income (<$50,000 as reference), race (White as reference), smoking status (never as reference), marital status (single as reference), body mass index (normal as reference), disease modifying therapy (non as reference)]. Values in bold considered significant.
(DOCX)

Supplementary Table 8 Regression models including interactions of within-person and between-person effects for HAD-D and HADS-A. RA = rheumatoid arthritis, IBD = inflammatory bowel disease, DEP/ANX = primary depression or anxiety, PCS = physical composite score; MCS = mental composite score, HADS = Hospital Anxiety and Depression Scale D = Depression, A = Anxiety, DFIS = daily fatigue impact scale, Zarm_leg = Physical functioning z-score which is an average for the z-score for the timed 25-foot walk and nine-hole peg test, SDMT = Symbol Digit Modalities Test. Unadjusted models include independent variables, no covariates. Adjusted models include independent variables + covariates; [age (continuous), age at symptom onset (continuous), gender (woman as reference), education (<high school as reference), income (<$50,000 as reference), race White as reference), smoking status (never as reference), marital status (single as reference), body mass index (normal as reference)]. Values in bold considered significant.
(DOCX)
