# Supplementary material for: Impact of depression and anxiety on health-related quality of life changes over time within individuals with rheumatoid arthritis or inflammatory bowel disease: A prospective Canadian cohort study
Source: PLoS One. 2026 May 28;21(5):e0349140. doi: 10.1371/journal.pone.0349140 (PMC13218540; doi:10.1371/journal.pone.0349140)
Supplement: Supplemental Table 1 — (DOCX) [file pone.0349140.s001.docx]

Supplemental Table 1. Disease Modifying Therapies used by rheumatoid arthritis and inflammatory bowel disease participants.

| **Disease** | **Steroids** | **Conventional disease modifying agents** | **Biologics and small molecules** |
| --- | --- | --- | --- |
| Rheumatoid Arthritis | Prednisone | Cyclosporin Hydroxychloroquine Minocycline  Sulfasalazine  Azathioprine  Cyclophosphamide  Leflunomide  Methotrexate  Mycophenolate | Infliximab,  Adalimumab  Etanercept  Certolizumab  Golimumab  Rituximab  Abatacept  Tocilizumab  Tofacitinib  Baricitinib |
| Inflammatory Bowel Disease | Prednisone Prednisolone Budesonide Hydrocortisone enema  Hydrocortisone acetate  Betamethasone | 5-ASA (oral, enemas or suppositories)  Mesalazine  Sulfasalazine  Mesalmine  Osalazine  Azathioprine  6-mercaptopurine  Methotrexate | Infliximab  Adalimumab  Golimumab  Ustekinumab  Vedolizumab |
