# Supplementary material for: Impact of depression and anxiety on health-related quality of life changes over time within individuals with rheumatoid arthritis or inflammatory bowel disease: A prospective Canadian cohort study
Source: PLoS One. 2026 May 28;21(5):e0349140. doi: 10.1371/journal.pone.0349140 (PMC13218540; doi:10.1371/journal.pone.0349140)
Supplement: Supplemental Table 2 — RA = rheumatoid arthritis, IBD = inflammatory bowel disease, DEP/ANX = primary depression or anxiety, SD = standard deviation, BMI = body mass index, MDD = major depressive disorder, HADS = Hospital Anxiety and Depression Scale D = depression A = anxiety, SDMT = Symbol Digit Modalities Test, 9HPT = nine hole peg test Physical functioning z-score which is an average of the z-score for the timed 25-foot walk and nine-hole peg test. 1p-value for comparison of RA versus DEP/ANX; 2 p-value for comparison of RA versus IBD; 3 p = value for comparison of IBD versus DEP/ANX; p value < 0.05 in bold considered significant. (DOCX) [file pone.0349140.s002.docx]

Supplemental Table 2 Patient comorbidity, symptoms, and function at baseline, stratified by disease group

| **Characteristic** | **RA** | **IBD** | **DEP/ANX** | **RA vs DEP/ANX**  **p-value^1^** | **RA vs IBD**  **p-value^2^** | **IBD vs DEP/ANX**  **p-value**^3^ |
| --- | --- | --- | --- | --- | --- | --- |
| N | 154 | 247 | 306 |  |  |  |
| **Number of comorbid conditions**, n (%) |  |  |  | **<0.001** | **<0.001** | **<0.001** |
| 0 | 20 (13) | 83 (33.6) | 87 (28.4) |  |  |  |
| 1 | 33 (21.4) | 58 (23.5) | 60 (19.6) |  |  |  |
| 2 | 25 (16.2) | 41 (16.6) | 61 (19.9) |  |  |  |
| ≥3 | 76 (49.4) | 65 (26.3) | 98 (32) |  |  |  |
| **Diagnoses of depression/anxiety disorders** |  |  |  |  |  |  |
| Lifetime MDD, n (%) | 58 (37.7) | 98 (39.7) | 251 (82) | **<0.001** | 0.688 | **<0.001** |
| Current MDD, n (%) | 17 (11) | 21 (8.5) | 85 (27.8) | **<0.001** | 0.399 | **<0.001** |
| Lifetime anxiety disorder, n (%) | 47 (30.5) | 65 (26.3) | 220 (71.9) | **<0.001** | 0.362 | **<0.001** |
| Current anxiety disorder, n (%) | 33 (21.4) | 47 (19) | 176 (57.5) | **<0.001** | 0.559 | **<0.001** |
| **HADS** |  |  |  |  |  |  |
| HADS-A, mean (SD) | 6.69 (3.93) | 6.34 (4.10) | 11.40 (4.04) | **<0.001** | 0.5242 | **<0.001** |
| HADS-D, mean (SD) | 4.90 (3.84) | 3.94 (3.67) | 8.17 (4.28) | **<0.001** | **<0.001** | **<0.001** |
| HADS-A ≥11, n (%) | 21 (13.73) | 41 (16.67) | 188 (61.44) | **<0.001** | 0.514 | **<0.001** |
| HADS-D ≥11, n (%) | 15 (9.74) | 16 (6.48) | 81 (26.56) | **<0.001** | **<0.01** | **<0.001** |
| **Daily Fatigue Impact Scale**, median (p25-p75) | 10 (4 -15) | 7.0 (2 -12) | 14.0 (7 -20) | **<0.001** | **<0.001** | **<0.001** |
| **SDMT z-score**, mean (SD) | -0.61 (1.02) | -0.13 (1.16) | -0.33 (1.24) | **<0.001** | **<0.01** | **<0.05** |
| **Timed 25 foot walk z-score** |  |  |  |  |  |  |
| Mean (SD) | -0.18 (0.91) | 0.29 (0.23) | 0.21 (0.33) | **<0.001** | **<0.001** | **0.001** |
| **9HPT z-score** |  |  |  |  |  |  |
| Mean (SD) | -0.45 (0.92) | 0.43 (0.82) | 0.25 (0.72) | **<0.001** | **<0.001** | **<0.01** |
| **Physical Functioning z-score**, mean (SD) | -0.31 (0.78) | 0.36 (0.46) | 0.22 (0.47) | **<0.001** | **<0.001** | **0.001** |

RA= rheumatoid arthritis, IBD = inflammatory bowel disease, DEP/ANX = primary depression or anxiety, SD = standard deviation, BMI = body mass index, MDD = major depressive disorder, HADS = Hospital Anxiety and Depression Scale D=depression A=anxiety, SDMT = Symbol Digit Modalities Test , 9HPT = nine hole peg test Physical functioning z-score which is an average of the z-score for the timed 25-foot walk and nine-hole peg test.

^1^p-value for comparison of RA versus DEP/ANX; ^2^ p-value for comparison of RA versus IBD; ^3^ p=value for comparison of IBD versus DEP/ANX; p value < 0.05 in bold considered significant.
