# Supplementary material for: Impact of depression and anxiety on health-related quality of life changes over time within individuals with rheumatoid arthritis or inflammatory bowel disease: A prospective Canadian cohort study
Source: PLoS One. 2026 May 28;21(5):e0349140. doi: 10.1371/journal.pone.0349140 (PMC13218540; doi:10.1371/journal.pone.0349140)
Supplement: Supplemental Table 3 — RA = rheumatoid arthritis, IBD = inflammatory bowel disease, DEP/ANX = primary depression or anxiety, SD = standard deviation, BMI = body mass index, HADS = Hospital Anxiety and Depression Scale D = depression A = anxiety, MDD = major depressive disorder, SDMT = Symbol Digit Modalities Test, 9HPT = nine hole peg test Physical functioning z-score which is an average of the z-score for the timed 25-foot walk and nine-hole peg test, DMT = disease modifying treatment. 1IBD completers vs non-completers, 2RA completers vs non-completers, 3DEP/ANX completers vs non-completers, p value < 0.05 in bold considered significant. (DOCX) [file pone.0349140.s003.docx]

Supplemental Table 3 Characteristics of study completers vs non-completers of all 4 visits stratified by disease group

| **Characteristic** | **IBD**  **Did not complete** | **IBD**  **Completed** | **P value^1^** | **RA**  **Did not complete** | **RA**  **Completed** | **P value^2^** | **DEP/ANX**  **Did not complete** | **DEP/ANX**  **Completed** | **P value^3^** |
| --- | --- | --- | --- | --- | --- | --- | --- | --- | --- |
| N | 33 | 214 |  | 25 | 129 |  | 60 | 246 |  |
| **Age**, yr mean (SD) | 46.08 (15.40) | 47.66 (14.73) | 0.57 | 56.48 (12.19) | 60.07 (11.51) | 0.16 | 40.38 (11.49) | 44.72 (13.16) | **0.02** |
| **Gender**, Woman n (%) | 26 (78.8) | 130 (60.8) | **0.05** | 20 (80) | 111 (86.1) | 0.54 | 41 (68.3) | 193 (78.5) | 0.13 |
| **Ethnicity**, n (%) |  |  | 0.20 |  |  | 0.58 |  |  | 0.47 |
| White | 25 (75.8) | 185 (86.9) |  |  | 99 (76.74) |  | 45 (75) | 200 (81.3) |  |
| Aboriginal | 3 (9.1) | 10 (4.7) |  |  | 9 (7.0) |  | 5 (8.3) | 17 (6.9) |  |
| Other | 5 (15.2) | 18 (8.5) |  | 6 (24) | 21 (16.3) |  | 10 (16.7) | 29 (11.8) |  |
| **Education**, n (%) |  |  | 0.14 |  |  | **0.01** |  |  | 0.12 |
| High school or less | 12 (36.4) | 64 (30.1) |  | 13 (52) | 37 (29.8) |  | 26 (43.3) | 75 (31.3) |  |
| College/Technical/Trade | 14 (42.4) | 67 (31.5) |  | 11 (44) | 50 (40.3) |  | 15 (25) | 90 (37.5) |  |
| University bachelors or higher | 7 (21.2) | 82 (38.5) |  | 1 (4) | 37 (29.8) |  | 19 (31.7) | 75 (31.3) |  |
| **Annual income**, n (%) |  |  | 0.95 |  |  | 0.43 |  |  | **0.04** |
| <$50,000 | 7 (21.2) | 51 (23.8) |  | 14 (56) | 56 (43.4) |  | 25 (41.7) | 117 (47.6) |  |
| >=$50,000 | 24 (72.7) | 147 (68.7) |  | 9 (36) | 64 (49.6) |  | 25 (41.7) | 114 (46.3) |  |
| I do not wish to answer | 2 (6.1) | 16 (7.5) |  | 2 (8) | 9 (7.0) |  | 10 (16.7) | 15 (6.1) |  |
| **Marital Status**, n (%) |  |  | 0.43 |  |  | 0.08 |  |  | 0.66 |
| Single/never married | 8 (24.2) | 48 (22.4) |  | 6 (24) | 13 (10.1) |  | 24 (40) | 86 (35) |  |
| Married/common law | 19 (57.6) | 141 (65.9) |  | 11 (44) | 82 (63.6) |  | 23 (38.3) | 110 (44.7) |  |
| Divorced/separated/widowed | 6 (18.2) | 25 (11.7) |  | 8 (32) | 34 (26.4) |  | 13 (21.7) | 50 (20.3) |  |
| **Smoking status**, n (%) |  |  | **0.03** |  |  | 0.60 |  |  | **0.04** |
| Never smoker | 13 (39.4) | 95 (44.4) |  | 8 (32) | 51 (39.5) |  | 26 (43.3) | 125 (50.8) |  |
| Past smoker | 9 (27.3) | 88 (41.1) |  | 12 (48) | 61 (47.3) |  | 14 (23.3) | 77 (31.3) |  |
| Current smoker | 11 (33.3) | 31 (14.5) |  | 5 (20) | 17 (13.2) |  | 20 (33.3) | 44 (17.9) |  |
| **Weight (kg), n (%)** |  |  | 0.86 |  |  | 1.00 |  |  | 0.78 |
| BMI <25 | 12 (36.4) | 84 (39.3) |  | 8 (32) | 45 (34.9) |  | 20 (33.3) | 75 (30.5) |  |
| BMI 25 to <30 | 11 (33.3) | 76 (35.5) |  | 8 (32) | 39 (30.2) |  | 17 (28.3) | 65 (26.4) |  |
| BMI ≥30 | 10 (30.3) | 54 (25.2) |  | 9 (36) | 45 (34.9) |  | 23 (38.3) | 106 (43.1) |  |
| **Number of comorbid conditions** n (%) |  |  | 0.80 |  |  | **0.02** |  |  | 0.15 |
| 0 | 10 (30.3) | 73 (34.1) |  | 6 (24) | 14 (10.9) |  | 24 (40) | 63 (25.6) |  |
| 1 | 10 (30.3) | 48 (22.4) |  | 6 (24) | 27 (20.9) |  | 12 (20) | 48 (19.5) |  |
| 2 | 5 (15.2) | 36 (16.8) |  | 0 (0) | 25 (19.4) |  | 9 (15) | 52 (21.1) |  |
| ≥3 | 8 (24.2) | 57 (26.6) |  | 13 (52) | 63 (48.8) |  | 15 (25) | 83 (33.7) |  |
| **Daily Fatigue Impact Scale** median (p25-p75) | 10.0  (7 -15) | 6.0  (2 -12) | **0.01** | 13  (5 -17) | 10  (4 -14) | 0.19 | 16.5  (13 -23) | 12.0  (7 -20) | **0.02** |
| **HADS** |  |  |  |  |  |  |  |  |  |
| HADS-A, mean (SD) | 7.67  (4.29) | 6.13  (4.04) | **0.05** | 7.36 (4.03) | 6.56  (3.91) | 0.36 | 11.70 (4.12) | 11.32  (4.03) | 0.52 |
| HADS-D, mean (SD) | 5.00  (3.95) | 3.78  (3.61) | 0.08 | 5.76 (3.84) | 4.74  (3.83) | 0.22 | 8.90 (4.19) | 7.99  (4.29) | 0.14 |
| HADS-A ≥11, n (%) | 10 (30.3) | 31 (14.6) | **0.04** | 4 (16) | 17 (13.3) | 0.75 | 39 (65) | 149 (60.6) | 0.56 |
| HADS-D ≥11, n (%) | 3 (9.1) | 13 (6.1) | 0.46 | 3 (12) | 12 (9.3) | 0.71 | 19 (31.7) | 62 (25.3) | 0.33 |
| **Diagnoses of depression/anxiety disorders** |  |  |  |  |  |  |  |  |  |
| Lifetime MDD, n (%) | 16 (48.5) | 82 (38.3) | 0.34 | 9 (36) | 49 (38) | 1.00 | 52 (86.7) | 199 (80.9) | 0.35 |
| Current MDD, n (%) | 6 (18.2) | 15 (7) | **0.04** | 4 (16) | 13 (10.1) | 0.48 | 20 (33.3) | 65 (26.4) | 0.33 |
| Lifetime anxiety disorder, n (%) | 7 (21.2) | 58 (27.1) | 0.53 | 10 (40) | 37 (28.7) | 0.34 | 43 (71.7) | 177 (72) | 1.00 |
| Current anxiety disorder, n (%) | 6 (18.2) | 41 (19.2) | 1.00 | 8 (32) | 25 (19.4) | 0.18 | 31 (51.7) | 145 (58.9) | 0.31 |
| **SDMT z-score**, mean (SD) | -0.37 (1.29) | -0.09 (1.13) | 0.20 | -1.06 (0.95) | -0.53  (1.02) | **0.02** | -0.54 (1.20) | -0.28  (1.25) | 0.14 |
| **Timed 25 foot walk z-score**, mean (SD) | 0.27 (0.20) | 0.29  (0.23) | 0.55 | -0.48 (1.75) | -0.12  (0.63) | 0.21 | 0.24 (0.26) | 0.20  (0.35) | 0.65 |
| **9HPT z-score**, mean (SD) | 0.41 (1.05) | 0.43  (0.78) | 0.92 | -0.50 (0.86) | -0.44  (0.93) | 0.75 | 0.18 (0.62) | 0.26  (0.74) | 0.41 |
| **Physical Functioning z-score**, mean (SD) | 0.34 (0.59) | 0.36  (0.44) | 0.84 | -0.49 (1.21) | -0.28  (0.67) | 0.41 | 0.21 (0.38) | 0.23  (0.49) | 0.77 |
| **Active disease**  **n (%)** | 15 (45.5) | 88 (42.3) | 0.85 | 13 (65) | 34 (27.6) | **<.01** |  |  |  |
| **Any Current DMT used,**  **n (%)** | 23 (69.7) | 162 (75.7) | 0.52 | 22 (88) | 122 (94.6) | 0.21 |  |  |  |

RA= rheumatoid arthritis, IBD = inflammatory bowel disease, DEP/ANX = primary depression or anxiety, SD = standard deviation, BMI = body mass index, HADS = Hospital Anxiety and Depression Scale D=depression A=anxiety, MDD = major depressive disorder, SDMT = Symbol Digit Modalities Test , 9HPT = nine hole peg test Physical functioning z-score which is an average of the z-score for the timed 25-foot walk and nine-hole peg test, BMI = body mass index, DMT = disease modifying treatment use.

^1^IBD completers vs non-completers, ^2^RA completers vs non-completers, ^3^DEP/ANX completers vs non-completers, p value < 0.05 in bold considered significant.
