# Supplementary material for: Impact of depression and anxiety on health-related quality of life changes over time within individuals with rheumatoid arthritis or inflammatory bowel disease: A prospective Canadian cohort study
Source: PLoS One. 2026 May 28;21(5):e0349140. doi: 10.1371/journal.pone.0349140 (PMC13218540; doi:10.1371/journal.pone.0349140)
Supplement: Supplemental Table 4 — RA = rheumatoid arthritis; IBD = inflammatory bowel disease, DEP/ANX = depression and anxiety, PCS = physical composite score; MCS = mental composite score, HADS = Hospital Anxiety and Depression Scale D = Depression, A = Anxiety, DFIS = daily fatigue impact scale, SDMT = Symbol Digit Modalities Test, NHP = nine hole peg test, SD = standard deviation; V1 = first (baseline) visit, V2 = second visit (first annual follow-up), V3 = third visit (second annual follow-up), V4 = forth annual follow-up) visit. (DOCX) [file pone.0349140.s004.docx]

Supplemental Table 4 Health- related quality of life, symptoms and function across study visits

| **Population** | **RA** | | | | **IBD** | | | | **DEP/ANX** | | | | |
| --- | --- | --- | --- | --- | --- | --- | --- | --- | --- | --- | --- | --- | --- |
| Variable | **V4** | **V2** | **V3** | **V4** | **V1** | **V2** | **V3** | **V4** | **V1** | **V2** | **V3** | **V4** |  |
| N | 154 | 149 | 134 | 134 | 247 | 239 | 225 | 216 | 306 | 285 | 267 | 249 |  |
| PCS-36,  mean (SD) | 30.7 (9.29) | 30.9 (9.49) | 31.0 (9.40) | 31.8 (9.65) | 37.6 (8.97) | 37.7 (8.90) | 38.1 (8.83) | 38.0 (9.25) | 35.0 (9.28) | 36.4 (9.59) | 35.9 (9.32) | 35.7 (9.18) |  |
| MCS-36,  mean (SD) | 37.6 (12.03) | 38.5 (12.52) | 37.6 (11.88) | 38.6 (11.69) | 38.8 (11.70) | 39.9 (11.91) | 39.2 (12.10) | 38.5 (12.16) | 25.8 (11.88) | 30.0 (13.04) | 28.4 (12.66) | 30.1 (12.75) |  |
| HADS-D,  mean (SD) | 4.9 (3.84) | 4.4 (3.59) | 4.9 (4.09) | 4.7 (3.76) | 3.9 (3.67) | 4.0 (3.65) | 3.9 (3.57) | 4.2 (3.73) | 8.2 (4.28) | 7.1 (4.47) | 7.0 (4.36) | 6.7 (4.49) |  |
| HADS-A,  mean (SD) | 6.7 (3.93) | 5.6 (3.98) | 6.0 (4.03) | 6.1 (4.08) | 6.3 (4.10) | 5.9 (4.21) | 6.6 (4.48) | 6.4 (4.33) | 11.4 (4.04) | 10.1 (4.51) | 10.4 (4.41) | 10.0 (4.53) |  |
| DFIS,  median (p25, p75) | 10  (4 -15) | 8  (3 -14) | 9  (3 -16) | 8  (3 -15) | 7  (2 -12) | 7  (2 -13) | 6  (2 -12) | 7  (2 -14) | 14  (7 -20) | 11  (6 -17) | 12  (6 -18) | 12  (6 -19) |  |
| SDMT z-score,  mean (SD) | -0.61 (1.02) | -0.54 (1.06) | -0.38 (1.15) | -0.39 (1.18) | -0.13 (1.16) | -0.04 (1.08) | 0.12 (1.23) | 0.18 (1.26) | -0.33 (1.24) | -0.21 (1.36) | -0.05 (1.31) | 0.11 (1.32) |  |
| Timed 25-foot walk Z-score,  mean (SD) | -0.18 (0.91) | -0.23 (0.79) | -0.21 (0.78) | -0.30 (0.92) | 0.29 (0.23) | 0.25 (0.26) | 0.23 (0.29) | 0.22 (0.30) | 0.21 (0.33) | 0.17 (0.33) | 0.08 (0.87) | 0.14 (0.40) |  |
| 9HPT z-score,  mean (SD) | -0.45 (0.92) | -0.47 (0.96) | -0.34 (0.99) | -0.37 (0.99) | 0.43 (0.82) | 0.36 (0.78) | 0.42 (0.85) | 0.47 (0.86) | 0.25 (0.72) | 0.24 (0.84) | 0.35 (0.76) | 0.39 (0.78) |  |
| Physical Functioning z-score,  mean (SD) | -0.31 (0.78) | -0.35 (0.78) | -0.27 (0.79) | -0.34 (0.82) | 0.36 (0.46) | 0.30 (0.46) | 0.33 (0.51) | 0.34 (0.51) | 0.22 (0.47) | 0.20 (0.55) | 0.21 (0.66) | 0.26 (0.53) |  |
| No. physical comorbidities | 2.71 (1.98) | 2.84 (1.99) | 3.06 (2.05) | 3.23 (2.08) | 1.68 (1.79) | 1.84 (1.88) | 1.89 (1.98) | 1.92 (1.96) | 2.00 (2.00) | 2.16 (2.07) | 2.28 (2.13) | 2.46 (2.20) |  |

RA = rheumatoid arthritis; IBD= inflammatory bowel disease, DEP/ANX= depression and anxiety, PCS=physical composite score; MCS = mental composite score, HADS = Hospital Anxiety and Depression Scale D = Depression, A = Anxiety, DFIS = daily fatigue impact scale, SDMT = Symbol Digit Modalities Test, NHP = nine hole peg test, SD = standard deviation; V1= first (baseline) visit, V2 = second visit (first annual follow-up) , V3= third visit (second annual follow-up), V4 = forth annual follow-up) visit.
