# Supplementary material for: Impact of depression and anxiety on health-related quality of life changes over time within individuals with rheumatoid arthritis or inflammatory bowel disease: A prospective Canadian cohort study
Source: PLoS One. 2026 May 28;21(5):e0349140. doi: 10.1371/journal.pone.0349140 (PMC13218540; doi:10.1371/journal.pone.0349140)
Supplement: Supplemental Table 5 — RA = rheumatoid arthritis, PCS = physical composite score; MCS = mental composite score, HADS = Hospital Anxiety and Depression Scale D = Depression, A = Anxiety, DFIS = daily fatigue impact scale, SDMT = Symbol Digit Modalities Test, DMT = disease modifying therapy, Zarm_leg = Z-score for physical function. Unadjusted models: Independent variables, no covariates; Adjusted models: Independent variables + covariates (age (continuous), age at symptom onset (continuous), gender (woman as reference), education (<high school as reference), income (<$50,000 as reference), race (White as reference), smoking status (never as reference), marital status (single as reference), Body mass index (normal as reference), disease modifying therapy). Values in bold considered significant. (DOCX) [file pone.0349140.s005.docx]

Supplemental Table 5 Rheumatoid Arthritis Regression models with continuous Hospital Anxiety and Depression Scale scores

| **Outcome** | **HRQOL -RA unadjusted models** | | **HRQOL -RA adjusted models** | |
| --- | --- | --- | --- | --- |
|  | **PCS-36**  **(95% CI)** | **MCS-36**  **(95% CI)** | **PCS-36**  **(95% CI)** | **MCS-36**  **(95% CI)** |
| N obs. Used in model | 569 | 568 | 518 | 517 |
| Between-person change in HADS-D | **-1.53**  **(-2.07, -0.99)** | **-2.19**  **(-2.57, -1.81)** | -0.45  (-0.95, 0.04) | **-2.08**  **(-2.45, -1.72)** |
| Within-person change in HADS-D | **-0.62**  **(-0.92, -0.31)** | **-0.82**  **(-1.18, -0.46)** | **-0.45**  **(-0.70, -0.20)** | **-0.78**  **(-1.15, -0.41)** |
| Between-person change in HADS-A | -0.08  (-0.59, 0.43) | **-0.65**  **(-1.03, -0.27)** | **0.62**  **(0.25, 1.00)** | **-0.67**  **(-1.02, -0.32)** |
| Within-person change in HADS-A | 0.03  (-0.20, 0.27) | **-0.86**  **(-1.18, -0.53)** | 0.16  (-0.06, 0.38) | **-0.75**  **(-1.11, -0.40)** |
| Between-person change in DFIS | **-0.89**  **(-1.01, -0.76)** | **-1.32**  **(-1.45, -1.20)** | **-0.62**  **(-0.85, -0.39)** |  |
| Within-person change in DFIS | **-0.32**  **(-0.43, -0.22)** | **-0.66**  **(-0.83, -0.48)** | **-0.24**  **(-0.35, -0.13)** |  |
| Between-person change in zarm_leg | **5.32**  **(3.34, 7.31)** | **4.59**  **(2.78, 6.41)** | **2.49**  **(0.85, 4.14)** | **1.37**  **(0.02, 2.72)** |
| Within-person change in zarm_leg | **2.72**  **(1.73, 3.72)** | **3.11**  **(0.74, 5.48)** | **2.08**  **(0.87, 3.29)** | **2.06**  **(0.28, 3.85)** |
| Between-person change in SDMT | **1.77**  **(0.39, 3.16)** | **2.35**  **(0.82, 3.88)** | **-0.93**  **(-1.86, 0.00)** | -0.11  (-1.04, 0.82) |
| Within-person change in SDMT | -0.33  (-1.13, 0.47) | 1.06  (-0.31, 2.43) | -0.67  (-1.38, 0.05) | 0.41  (-0.84, 1.66) |
| Between-person change in disease activity | **-13.16**  **(-16.16, -10.17)** | **-13.91**  **(-18.45, -9.37)** | **-3.51**  **(-5.82, -1.20)** | -0.64  (-3.22, 1.94) |
| Within-person change in disease activity | **-2.23**  **(-3.39, -1.07)** | **-1.89**  **(-3.55, -0.23)** | **-1.67**  **(-2.79, -0.54)** | **-1.82**  **(-3.48, -0.16)** |
| Between-person change in no. comorbidities | **-1.33**  **(-1.95, -0.71)** | -0.68  (-1.49, 0.13) | **-0.87**  **(-1.30, -0.43)** | -0.24  (-0.69, 0.21) |
| Within-person change in no. comorbidities | -0.30  (-1.17, 0.56) | 0.87  (-0.55, 2.29) | -0.03  (-0.80, 0.74) | 0.82  (-0.50, 2.13) |

RA=rheumatoid arthritis, PCS=physical composite score; MCS = mental composite score, HADS = Hospital Anxiety and Depression Scale D = Depression, A = Anxiety, DFIS = daily fatigue impact scale, SDMT = Symbol Digit Modalities Test, DMT= disease modifying therapy, Zarm_leg = Z-score for physical function

Unadjusted models: Independent variables, no covariates; Adjusted models: Independent variables + covariates (age (continuous), age at symptom onset (continuous), gender (woman as reference), education (< high school as reference), income (<$50,000 as reference), race (White as reference), smoking status (never as reference), marital status (single as reference), Body mass index (normal as reference), disease modifying therapy)

Values in bold considered significant
