# Supplementary material for: Impact of depression and anxiety on health-related quality of life changes over time within individuals with rheumatoid arthritis or inflammatory bowel disease: A prospective Canadian cohort study
Source: PLoS One. 2026 May 28;21(5):e0349140. doi: 10.1371/journal.pone.0349140 (PMC13218540; doi:10.1371/journal.pone.0349140)
Supplement: Supplemental Table 6 — IBD = inflammatory bowel disease, CD = Crohn’s disease, UC = ulcerative colitis, PCS = physical composite score; MCS = mental composite score, HADS = Hospital Anxiety and Depression Scale D = Depression, A = Anxiety, DFIS = daily fatigue impact scale, SDMT = Symbol Digit Modalities Test. Unadjusted models: Independent variables, no covariates; Adjusted models include independent variables + covariates [age (continuous), age at symptom onset (continuous), gender(woman as reference), education (<high school as reference), income (<$50,000 as reference), race (White as reference), smoking status (never as reference), marital status (single as reference), body mass index (normal as reference), disease modifying therapy (none as reference)]. Values in bold considered significant. (DOCX) [file pone.0349140.s006.docx]

Supplemental Table 6 Inflammatory Bowel Disease regression models with continuous Hospital Anxiety and Depression Scale scores

|  | **HRQOL -IBD unadjusted models** | | **HRQOL -IBD adjusted models** | |
| --- | --- | --- | --- | --- |
| **Outcome** | **PCS-36** | **MCS-36** | **PCS-36** | **MCS-36** |
|  | **(95% CI)** | **(95% CI)** | **(95% CI)** | **(95% CI)** |
| N obs. Used in model | 924 | 924 | 844 | 844 |
| Between-person change in HADS-D | **-1.43**  **(-1.80, -1.06)** | **-2.08**  **(-2.40, -1.75)** | -0.31  (-0.65, 0.04) | **-1.97**  **(-2.30, -1.64)** |
| Within-person change in HADS-D | **-0.64**  **(-0.82, -0.45)** | **-1.45**  **(-1.70, -1.20)** | **-0.28**  **(-0.48, -0.08)** | **-1.45**  **(-1.72, -1.17)** |
| Between-person change in HADS-A | -0.25  (-0.57, 0.07) | **-0.74**  **(-1.03, -0.45)** | -0.03  (-0.28, 0.21) | **-0.66**  **(-0.94, -0.38)** |
| Within-person change in HADS-A | **-0.18**  **(-0.34, -0.01)** | **-0.74**  **(-0.98, -0.49)** | -0.12  (-0.28, 0.04) | **-0.75**  **(-1.01, -0.49)** |
| Between-person change in DFIS | **-0.97**  **(-1.10, -0.85)** | **-1.27**  **(-1.41, -1.13)** | **-0.58**  **(-0.72, -0.43)** |  |
| Within-person change in DFIS | **-0.42**  **(-0.49, -0.34)** | **-0.78**  **(-0.91, -0.64)** | **-0.30**  **(-0.37, -0.22)** |  |
| Between-person change in zarm_leg | **6.24**  **(4.33, 8.15)** | **4.62**  **(2.26, 6.97)** | 1.13  (-0.69, 2.95) | **2.10**  **(-0.03, 4.22)** |
| Within-person change in zarm_leg | **3.59**  **(1.87, 5.30)** | 2.17  (-0.34, 4.68) | **2.20**  **(0.60, 3.80)** | 0.46  (-1.74, 2.67) |
| Between-person change in SDMT | **2.11**  **(1.19, 3.02)** | **1.51**  **(0.31, 2.71)** | 0.08  (-0.52, 0.68) | -0.63  (-1.42, 0.16) |
| Within-person change in SDMT | 0.36  (-0.14, 0.85) | 0.07  (-0.71, 0.84) | 0.07  (-0.37, 0.50) | -0.13  (-0.81, 0.55) |
| Between-person change in disease activity | **-12.75**  **(-14.90, -10.61)** | **-11.77**  **(-15.02, -8.52)** | **-5.92**  **(-7.59, -4.26)** | **-2.24**  **(-4.22, -0.25)** |
| Within-person change in disease activity | **-3.47**  **(-4.53, -2.40)** | **-3.54**  **(-5.27, -1.82)** | **-2.74**  **(-3.62, -1.85)** | **-2.04**  **(-3.43, -0.65)** |
| Between-person change in no. comorbidities | **-1.73**  **(-2.24, -1.23)** | **-0.80**  **(-1.44, -0.15)** | **-1.03**  **(-1.43, -0.64)** | -0.40  (-0.89, 0.09) |
| Within-person change in no. comorbidities | 0.18  (-0.73, 1.10) | 0.16  (-1.02, 1.34) | 0.21  (-0.42, 0.85) | 0.41  (-0.55, 1.38) |
| IBD disease type (CD vs UC) | **-** | **-** | -0.72  (-1.81, 0.37) | -0.07  (-1.47, 1.32) |

IBD= inflammatory bowel disease, CD=crohn’s disease, UC = ulcerative colitis, PCS=physical composite score; MCS = mental composite score, HADS = Hospital Anxiety and Depression Scale D = Depression, A = Anxiety, DFIS = daily fatigue impact scale, SDMT = Symbol Digit Modalities Test. Unadjusted models: Independent variables, no covariates; Adjusted models include independent variables + covariates [age (continuous), age at symptom onset (continuous), gender (woman as reference), education (< high school as reference), income (< $50,000 as reference), race (White as reference), smoking status (never as reference), marital status (single as reference), body mass index (normal as reference), disease modifying therapy (none as reference)] Values in bold considered significant.
