# Supplementary material for: Impact of depression and anxiety on health-related quality of life changes over time within individuals with rheumatoid arthritis or inflammatory bowel disease: A prospective Canadian cohort study
Source: PLoS One. 2026 May 28;21(5):e0349140. doi: 10.1371/journal.pone.0349140 (PMC13218540; doi:10.1371/journal.pone.0349140)
Supplement: Supplemental Table 7 — PCS = physical composite score; MCS = mental composite score, HADS = Hospital Anxiety and Depression Scale D = Depression, A = Anxiety, DFIS = daily fatigue impact scale, Zarm_leg = Physical functioning z-score which is an average for the z-score for the timed 25-foot walk and nine-hole peg test, SDMT = Symbol Digit Modalities Test Unadjusted models include independent variables, no covariates. Adjusted models include independent variables + covariates [age (continuous), age at symptom onset (continuous), gender (woman as reference), education (<high school as reference), income (<$50,000 as reference), race (White as reference), smoking status (never as reference), marital status (single as reference), body mass index (normal as reference), disease modifying therapy (non as reference)]. Values in bold considered significant. (DOCX) [file pone.0349140.s007.docx]

Supplemental Table 7 Depression and Anxiety Regression models for contributors to health-related quality of life

|  | **Unadjusted** | | **Adjusted*** | |
| --- | --- | --- | --- | --- |
| **Outcome** | **PCS-36** | **MCS-36** | **PCS-36** | **MCS-36** |
|  | **(95% CI)** | **(95% CI)** | **(95% CI)** | **(95% CI)** |
| N obs. Used in model |  |  | 924 | 922 |
| Between-person change in HADS-D ≥11 | **-11.34**  **(-14.03, -8.65)** | **-22.04**  **(-24.34, -19.73)** | -2.58  (-5.15, -0.011) | **-16.04**  **(-18.31, -13.77)** |
| Within-person change in HADS-D ≥11 | **-2.07**  **(-3.39, -0.84)** | **-9.58**  **(-11.7, -7.46)** | -0.72  (-1.83, 0.38) | **-8.77**  **(-10.57, -6.77)** |
| Between-person change in HADS-A ≥11 | **-6.25**  **(-8.61, -3.89)** | **-16.79**  **(-19.34, -14.24)** | -0.18  (-2.18, 1.82) | **-10.59**  **(-12.91, -8.27)** |
| Within-person change in HADS-A ≥11 | **-1.41**  **(-2.30, -0.52)** | **-6.29**  **(-8.12, -4.45)** | -0.73  (-1.53, 0.067) | **-5.79**  **(-7.46, -4.13)** |
| Between-person change in DFIS | **-0.76**  **(-0.87, -0.66)** | **-1.16**  **(-1.27, -1.05)** | -0.56  (-0.69, -0.44) |  |
| Within-person change in DFIS | **-0.33**  **(-0.39, -0.27)** | **-0.70**  **(-0.83, -0.58)** | **-0.32**  **(-0.39, -0.25)** |  |
| Between-person change in zarm_leg | **7.95**  **(5.59, 10.32)** | **4.57**  **(1.30, 7.83)** | **3.74**  **(1.96, 5.51)** | 0.68  (-2.12, 3.49) |
| Within-person change in zarm_leg | 0.31  (-0.77, 1.40) | 1.17  (-0.66, 3.00) | 0.049  (-0.97, 1.07) | 0.15  (-1.44, 1.75) |
| Between-person change in SDMT | **2.03**  **(1.32, 2.74)** | **1.09**  **(0.18, 2.01)** | -0.042 (-0.74, 0.65) | 0.22  (-0.55, 0.98) |
| Within-person change in SDMT | -0.35  (-0.95, 0.24) | **1.33**  **(0.12, 2.55)** | -0.59  (-1.11, -0.079) | 0.61  (-0.42, 1.65) |
| Between-person change in no. comorbidities | **-1.77**  **(-2.16, -1.39)** | -0.34  (-0.91, 0.24) | -1.20  (-1.58, -0.83) | **-0.30**  **(-0.78, 0.18)** |
| Within-person change in no. comorbidities | **-0.78**  **(-1.45, -0.12)** | 0.28  (-0.84, 1.40) | **-0.69**  **(-1.32, -0.057)** | -0.049  (-1.03, 0.93) |

PCS=physical composite score; MCS = mental composite score, HADS = Hospital Anxiety and Depression Scale D = Depression, A = Anxiety, DFIS = daily fatigue impact scale, Zarm_leg = Physical functioning z-score which is an average for the z-score for the timed 25-foot walk and nine-hole peg test, SDMT = Symbol Digit Modalities Test Unadjusted models include independent variables, no covariates. Adjusted models include independent variables + covariates (age (continuous), age at symptom onset (continuous), gender (woman as reference), education (< high school as reference), income (< $50,000 as reference), race (White as reference), smoking status (never as reference), marital status (single as reference), body mass index (normal as reference), disease modifying therapy (non as reference).] Values in bold considered significant
