# Supplementary material for: Impact of depression and anxiety on health-related quality of life changes over time within individuals with rheumatoid arthritis or inflammatory bowel disease: A prospective Canadian cohort study
Source: PLoS One. 2026 May 28;21(5):e0349140. doi: 10.1371/journal.pone.0349140 (PMC13218540; doi:10.1371/journal.pone.0349140)
Supplement: Supplementary Table 8 — RA = rheumatoid arthritis, IBD = inflammatory bowel disease, DEP/ANX = primary depression or anxiety, PCS = physical composite score; MCS = mental composite score, HADS = Hospital Anxiety and Depression Scale D = Depression, A = Anxiety, DFIS = daily fatigue impact scale, Zarm_leg = Physical functioning z-score which is an average for the z-score for the timed 25-foot walk and nine-hole peg test, SDMT = Symbol Digit Modalities Test. Unadjusted models include independent variables, no covariates. Adjusted models include independent variables + covariates; [age (continuous), age at symptom onset (continuous), gender (woman as reference), education (<high school as reference), income (<$50,000 as reference), race White as reference), smoking status (never as reference), marital status (single as reference), body mass index (normal as reference)]. Values in bold considered significant. (DOCX) [file pone.0349140.s008.docx]

Supplemental Table 8: Regression models including interactions of within-person and between-person effects for HAD-D and HADS-A

|  |  |  |  |  |  |  |
| --- | --- | --- | --- | --- | --- | --- |
| **Observation** | **RA** | **RA** | **IBD** | **IBD** | **RA IBD DEP/ANX** | **RA IBD DEP/ANX** |
|  | **PCS-36** | **PCS-36** | **PCS-36** | **MCS-36** | **PCS-36** | **MCS-35** |
| N obs. Used in model | 518 | 517 | 844 | 844 | 2464 | 2462 |
| Between-person change in HADS-D | -0.44  (-0.94 , 0.05) | **-2.08**  **(-2.44 , -1.72)** | -0.31  (-0.65 , 0.03) | **-1.98**  **(-2.31 , -1.65)** | **-0.48**  **(-0.67 , -0.29)** | **-1.93**  **(-2.10 , -1.76)** |
| Within-person change in HADS-D | **-1.05**  **(-1.62 , -0.49)** | **-1.06**  **(-1.80 , -0.32)** | **-0.38**  **(-0.72 , -0.04)** | **-1.85**  **(-2.41, -1.29)** | **-0.67**  **(-0.89, -0.45)** | **-2.06**  **(-2.42 , -1.69)** |
| *Between-pc in HADS-D * within pc in HADS-D* | **0.08**  **(0.01 , 0.15)** | 0.04  (-0.05 , 0.13) | 0.02  (-0.04 , 0.07) | 0.07  (-0.01 , 0.15) | **0.05**  **(0.02 , 0.08)** | **0.10**  **(0.05 , 0.14)** |
| Between-person change in HADS-A | **0.63**  **(0.25 , 1.00)** | -0.67  (-1.02 , -0.32) | -0.03  (-0.27 , 0.21) | **-0.66**  **(-0.93 , -0.38)** | 0.07  (-0.09 , 0.22) | **-0.73**  **(-0.90 , -0.57)** |
| Within-person change in HADS-A | 0.07  (-0.41 , 0.56) | -0.13  (-0.91 , 0.64) | 0.10  (-0.27 , 0.46) | -0.38  (-0.95 , 0.20) | 0.08  (-0.14 , 0.31) | **-0.67**  **(-1.06 , -0.28)** |
| *Between-pc in HADS-A * within pc in HADS-A* | 0.01  (-0.04 , 0.07) | -0.08  (-0.17 , 0.01) | -0.03  (-0.07 , 0.02) | -0.05 (-0.11 , 0.02) | -0.010  (-0.03 , 0.01) | -0.01  (-0.05 , 0.02) |
| Between-person change in DFIS | **-0.63**  **(-0.86 , -0.39)** |  | **-0.58**  **(-0.73 , -0.43)** |  | **-0.57**  **(-0.66 , -0.48)** |  |
| Within-person change in DFIS | **-0.24**  **(-0.34 , -0.13)** |  | **-0.29**  **(-0.37 , -0.22)** |  | **-0.28**  **(-0.33 , -0.23)** |  |
| Between-person change in zarm_leg | **2.48**  **(0.85 , 4.10)** | **1.37**  **(0.02 , 2.72)** | 1.14  (-0.68 , 2.96) | **2.11**  **(-0.01 , 4.24)** | **2.97**  **(1.84 , 4.10)** | **1.76**  **(0.65 , 2.86)** |
| Within-person change in zarm_leg | **2.01**  **(0.88 , 3.15)** | **1.92**  **(0.23 , 3.61)** | **2.21**  **(0.61 , 3.81)** | 0.49  (-1.72 , 2.70) | **1.32**  **(0.52 , 2.11)** | 0.60  (-0.43 , 1.64) |
| Between-person change in SDMT | **-0.93**  **(-1.86 , 0.00)** | -0.10  (-1.04 , 0.84) | 0.08  (-0.52 , 0.68) | -0.64  (-1.42 , 0.15) | -0.24  (-0.70 , 0.21) | **-0.45**  **(-0.88 , -0.03)** |
| Within-person change in SDMT | -0.70  (-1.41 , 0.02) | 0.39  (-0.86 , 1.64) | 0.07  (-0.36 , 0.51) | -0.13  (-0.81 , 0.56) | **-0.38**  **(-0.70 , -0.07)** | 0.19  (-0.31 , 0.69) |
| Between-person change in disease activity | **-3.47**  **(-5.79 , -1.15)** | -0.64  (-3.25 , 1.96) | **-5.92**  **(-7.58 , -4.26)** | **-2.25**  **(-4.23 , -0.27)** |  |  |
| Within-person change in disease activity ** | **-1.54**  **(-2.66 , -0.42)** | **-1.74**  **(-3.34 , -0.14)** | **-2.79**  **(-3.66 , -1.91)** | **-2.14**  **(-3.52 , -0.76)** |  |  |
| Between-person change in no. comorbidities | **-0.87 (-1.31 , -0.44)** | -0.24  (-0.69 , 0.21) | **-1.04**  **(-1.43 , -0.64)** | -0.41  (-0.90 , 0.08) | **-1.07**  **(-1.32 , -0.82)** | -0.22  (-0.50 , 0.05) |
| Within-person change in no. comorbidities | -0.09 (-0.86 , 0.67) | 0.91  (-0.37 , 2.19) | 0.21 (-0.42 , 0.84) | 0.43  (-0.53 , 1.39) | -0.31  (-0.71 , 0.09) | 0.09  (-0.52 , 0.69) |
| IBD disease type (CD vs UC) |  |  | -0.72 (-1.81 , 0.37) | -0.08  (-1.47 , 1.31) |  |  |
| Disease Cohort |  |  |  |  |  |  |
| RA |  |  |  |  | ref | Ref |
| Depression/Anxiety disorder |  |  |  |  | **5.60**  **(4.30 , 6.91)** | -1.26  (-2.59 , 0.06) |
| IBD |  |  |  |  | **2.71**  **(1.49 , 3.92)** | -0.71  (-1.95 , 0.53) |

RA= rheumatoid arthritis, IBD = inflammatory bowel disease, DEP/ANX = primary depression or anxiety, PCS=physical composite score; MCS = mental composite score, HADS = Hospital Anxiety and Depression Scale D = Depression, A = Anxiety, DFIS = daily fatigue impact scale, Zarm_leg = Physical functioning z-score which is an average for the z-score for the timed 25-foot walk and nine-hole peg test, SDMT = Symbol Digit Modalities Test.

Unadjusted models include independent variables, no covariates. Adjusted models include independent variables + covariates [age (continuous), age at symptom onset (continuous), gender (woman as reference), education (< high school as reference), income (< $50,000 as reference), race (White as reference), smoking status (never as reference), marital status (single as reference), body mass index (normal as reference), disease modifying therapy (none as reference)]. Values in bold considered significant
